# Artificial intelligence for reducing the radiation burden of medical imaging for the diagnosis of coronavirus disease

**DOI:** 10.1140/epjp/s13360-023-03745-4

**Published:** 2023-05-08

**Authors:** Jiaxi Hu, Stavroula Mougiakakou, Song Xue, Ali Afshar-Oromieh, Wolf Hautz, Andreas Christe, Raphael Sznitman, Axel Rominger, Lukas Ebner, Kuangyu Shi

**Affiliations:** 1 Department of Nuclear Medicine, Inselspital, Bern University Hospital, University of Bern, Freiburgstrasse 18, 3010 Bern, Switzerland; 2 ARTORG Center for Biomedical Engineering Research, University of Bern, Murtenstrasse 50, 3008 Bern, Switzerland; 3 Department of University Emergency Center of Inselspital, University of Bern, Freiburgstrasse 15, 3010 Bern, Switzerland; 4 Department of Radiology, Inselspital, Bern University Hospital, University of Bern, 3012 Bern, Switzerland

## Abstract

Medical imaging has been intensively employed in screening, diagnosis and monitoring during the COVID-19 pandemic. With the improvement of RT–PCR and rapid inspection technologies, the diagnostic references have shifted. Current recommendations tend to limit the application of medical imaging in the acute setting. Nevertheless, efficient and complementary values of medical imaging have been recognized at the beginning of the pandemic when facing unknown infectious diseases and a lack of sufficient diagnostic tools. Optimizing medical imaging for pandemics may still have encouraging implications for future public health, especially for long-lasting post-COVID-19 syndrome theranostics. A critical concern for the application of medical imaging is the increased radiation burden, particularly when medical imaging is used for screening and rapid containment purposes. Emerging artificial intelligence (AI) technology provides the opportunity to reduce the radiation burden while maintaining diagnostic quality. This review summarizes the current AI research on dose reduction for medical imaging, and the retrospective identification of their potential in COVID-19 may still have positive implications for future public health.

## Introduction

The World Health Organization has characterized the outbreak of COVID-19 as a pandemic, and the whole world is suffering from this global health, social and economic crisis. Because of the fast replication in the pharynx and lungs, severe acute respiratory syndrome coronavirus (SARS-COV) spreads very fast and has a relatively high mortality rate [1, 2]. A key issue during the outbreak is to contain the epidemic and the rapid and proper management of patients. A rapid diagnosis and screening tool is necessary to treat patients in time, prevent the spread of virus carriers and rule out unnecessary medical occupations. Previously, at the first surge of the pandemic, medical imaging was widely applied as a complementary tool in the screening and diagnosis of COVID-19 patients [3, 4]. Chest radiographic and/or computed tomography imaging is an efficient adjunct to real-time reverse transcription-polymerase chain reaction (RT-PCR) for COVID [5, 6]. With the improvement of accuracy and extended availability of various virus tests, the role of imaging is constantly being weakened. Digital screening surveillance modalities remain irreplaceable for COVID-19 patients to monitor respiratory disease afterward, as well as for PCR-negative patients but with typical clinical symptoms to recheck their lung condition [7]. However, concerns about the radiation burden were also raised in the screening for infectious diseases [8]. A consensus has been reached to minimize the radiation burden on the population in the management of COVID-19 or other epidemic diseases. Although the space for medical imaging in COVID-19 diagnosis is reduced, it may still be worth discussing the potential of AI technology in dose reduction for COVID-19 imaging. This review aims to summarize the current AI research in dose reduction for medical imaging and retrospectively discusses their potential in the assistance of the COVID-19 pandemic and post-pandemic management.

## Complementarity of medical imaging in COVID-19 diagnosis

The virus nucleic acid RT-PCR is the current gold standard for the diagnosis of a coronavirus infection [2]. Considering the pathogenicity of SARS-Co-2 and the sensitive nature of the current pandemic situation, different protocols have been put into adopted in the same time. The available RT-PCR protocols with differing sensitivity/specificity for COVID-19 diagnosis at the surge of the pandemic have been summarized in studies [9, 10], and researchers have reached a consensus that the recommended RT-PCR requires an additional complementarity. It has several limitations, such as difficulties in sample collection and transfer, sample contamination, limited protocol supplies and rigorous requirements for laboratory settings [11–16], which could cause an even greater challenge and stressful setting by creating a larger false-negative population in a pandemic situation. A retrospective study that investigated 1014 COVID-19 patients showed that only 59 percent had positive RT-PCR results [11]. In addition, test results are not immediately available but take between 5 h and several days. Even though the rapid RT-PCR protocol has been adopted in clinical practice, in addition to the high false-negative rates and variabilities in test techniques, there are evidence that show the possibility of undetectable PCR test [17–19].

In contrast, the results of imaging procedures are immediately available, thereby leading to much faster isolation and treatment of patients. At the beginning of the pandemic, imaging has been used as a complementary tool in the diagnosis of COVID-19, particularly in situations with a shortage of capacity for RT-PCR tests or limited RT-PCR sensitivity due to errors in sample collection or processing [10, 13, 20]. In early diagnostic practice in severe COVID-19 epidemics in China, imaging was considered to have a higher sensitivity than RT-PCR. Studies have shown that CT identified a large number of COVID-19 patients with initial negative RT-PCR results [11, 20, 21]. Furthermore, the effect of imaging management tools in COVID-19 diagnosis was proven to be pragmatic in worldwide [22, 23]. The complementary imaging screening approach at that time helped to contain the rapid spread of COVID-19 infections. The cost of sterilized screening has also limited the widely and density performed. The chest radiography and CT still be meaningful in monitoring severe lung involvement, particularly in the ICU admission environment. Meanwhile, the main concern of persistent symptoms of variable severity still calls for long-term CT imaging-based diagnosis, a subset of patient will have persistent chest imaging abnormalities after them recovering from COVID-19 [24]. To meet the challenge of post COVID-19 care, the healthcare system put emphasis on long-term medical imaging scanning to reveal the symptoms evolution, recover process and the delayed pathology screening appearance of post-acute COVID-19 [25, 26]. The relative contributions of long-term CT changes after COVID-19, including inflammatory evaluation, delayed pulmonary fibrotic and ventilation disorder, are currently needed for monitoring disease progression and treatment management. In formulating the response of the healthcare system to the COVID-19 pandemic, the radiation burden must be uncovered to show the actual toll of the chronic consequences of SARS-CoV-2 infection, especially for long COVID-19 [27]. Therefore, the radiation burden remaining in the long-term monitoring of COVID-19 pneumonia and post COVID-19 chest abnormalities after PCR tests becomes negative, which is a call for an applicable dose-reduction tool in COVID-19 management. After careful considering the screening, diagnosis and monitoring benefits, their contribution may still needed in dealing with future public health crises.

Although not often used in emergency settings, PET has also shown complementary value in the diagnosis of COVID-19. Cases of COVID-19 have been reported with negative CT and RT–PCR results but demonstrated a positive PET examination [28]. Furthermore, there is growing attention on underestimated evidence of long COVID [29, 30], which shows about 38 percent of discharged patients present lasting abnormality in chest radiographs [31], and the pulmonary fibrosis was detected in thin-section CT in 39 percent of patients after hospital discharge [32]. Furthermore, multimodal application of immunologic PET/CT [33] may provide a promising tool to understand multiorgan involvement of COVID-19. Radiolabeling of inflammatory cells for PET imaging [34] for the identification of immunologic “checkpoints” may be of interest for targeted drug development. In addition to being used in various stages of disease to guide management, PET/CT can be used to select and monitor patients with immunologic interventions.

**Fig. 1:**
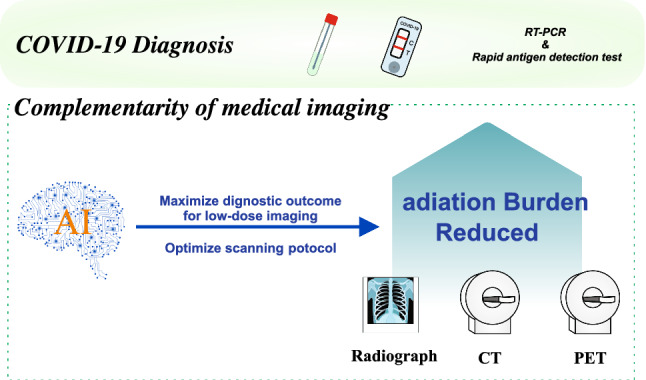
The main concept of this reducing radiation burdon in COVID-19 diagnosis

We here to discuss the role of AI in reducing radiation burden in medical imaging based COVID-19 diagnosis, the main concept shown in Fig. 1.

## AI in COVID-19

Recent advances in artificial intelligence (AI) and specifically in deep learning have led to substantial changes in medical imaging [35–37]. It replicates and extends human powers of perception for information from data (e.g., images) and may surpass human performance in some situations [5, 38]. Thus, it has brought record-breaking performance in many applications, such as image recognition, robotics and self-driving cars [35]. It has already demonstrated advantages in computerized diagnosis on medical imaging, such as differential diagnosis of skin cancer [39] or diabetic retinopathy [40]. The application of deep learning has been extended to image enhancement [41]and cross-modality synthesis [42]. Generative adversarial networks (GANs), which can mimic any data distribution, are another exciting advancement in deep learning [43]. AI has demonstrated its potential in assisting the diagnosis of COVID-19, and its corresponding application in computer-aided diagnosis has successfully improved diagnostic efficiency in many applications. The current literature mainly focuses on revealing AI’s potentiality in assisted diagnosis [44–48], and some in segmentation [49, 50]. More specifically, to address the difficulty of emerging false positives in CT findings, Lin et al. trained a 2D convolutional neural networking (CNN) method to differentiate COVID-19 from community-acquired pneumonia and non-pneumonia [51]. Harrison and Robin et al. established an AI system for differentiating COVID-19 and non-coronavirus pneumonia, which improved radiologists’ performance in accurately diagnosing COVID-19 [48]. As the standardized assessment of pulmonary involvement of COVID-19 on chest CT images, COVID-19 Reporting and Data System (Co-RADS), has been developed [52], the related AI-assisted automated assessment tool has been developed and presents high diagnostic performance with a receiver operating characteristic (AUC) curve of 0.95 in the internal cohort and an AUC of 0.88 in the external cohort [53]. It is noteworthy that this Co-RADS AI system is based on the categories directly interpretable by radiologists, which provides an excellent example for future investigation in integrating the AI method in clinical protocols. Although AI has demonstrated potential value in COVID diagnosis, pitfalls of AI have been reported, and the developed AI methods lack potential clinical use due to methodological flaws and/or underlying biases [54].

## AI to enhence CT and radiograph

Chest radiography and chest CT have been reported as critical medical imaging technologies to identify and investigate suspected patients with COVID-19 in the initial diagnostic screen and to monitor infection progression during follow-up observation in clinical practice. An increasing number of AI-based models have been developed for the diagnosis and prognosis of COVID-19 using both CT scans and radiographs. These models utilize AI-based methods to extract COVID-19 relevant features, such as lung patterns and disease-specific manifestations [51, 52, 55–57]. A retrospective study conducted in a tertiary hospital in the United States has reported that AI-read chest radiographs could potentially have comparable prognostic performance to CT scans [55]. Recent studies have shown that deep learning on chest CT can accurately detect COVID-19 and differentiate it from community-acquired pneumonia and other lung diseases [51], while many companies have offered software to support physicians using AI tools for the diagnosis of COVID-19 from CT. Furthermore, several open access databases are boosting the research in this direction. An AI-empowered medical workflow can significantly help automate scanning and diagnosis procedures, including lesion detection, segmentation, pneumonia classification, severity assessment and prognosis estimation, which assist COVID-19 pneumonia screening in characterization for disease diagnosis, tracking, and prognosis [56–58], and error reduction is one of the keys in AI-driven diagnostic tools to facilitate imaging interpretation. Although a number of studies show that the AI-assist diagnosis protocol provides excellent performance, this article surveys publications directly related to alleviating the radiation burden.

## AI to reduce the dose of CT

Despite the role of imaging in the diagnosis and control of COVID-19, the specificity of imaging in the diagnosis of COVID-19 is still generally low. Radiation exposure remains a critical concern in practice. Although conventional chest radiograph (CXR) imaging remains the most common first-line imaging tool for chest assessment and is most appropriate for infection surveillance, it is only suitable for pretest and has until now limited value in the differential diagnosis of COVID-19 [59].

As chest CT scan utilization is the main cause of radiation exposure buildup during the COVID-19 pandemic, Low-dose computed tomography (LDCT) became the advisable management decision, and together with AI-based tools, it has significant advances in clinical management. LDCT was first introduced in the 1990 s for lung cancer-related screening [60], which is recommended in COVID-19 diagnosis with up to an 8-fold dose reduction [61]. Taking advantage of the ability of AI to quantify information from images and its superior capability in recognizing complex patterns in images compared to humans, the developed AI algorithm for computer-aided diagnosis on standard CT has extended to computer-aided diagnosis on LDCT. The main concern regarding dose reduction applications can compromise image quality and corresponding diagnostic efficiency, which can lead to data loss in the procedure, resulting in decreased image quality and unpredictable artifacts. Therefore, there are studies working on AI based denoising in LDCT and thus help limit radiation exposure hazards [62, 63]. Following the dose principle of ALARA (as low as reasonably achievable), deep learning-based reconstruction (DLR) techniques can improve image quality compared to iterative reconstruction (IR), where the root-mean-square error (RMSE) was limited within (−0.23, 0.47), and thus enable CT radiation dose reduction [64, 65]. With the help of AI technologies, especially deep learning algorithms, various LDCT imaging reconstruction solutions have been developed, such as adaptive statistical iterative reconstruction (ASIR) and novel model-based iterative reconstruction algorithms (MBIR) [66] and the quadratic autoencoder (Q-AE) method [67], which show encouraging outcomes in terms of noise reduction abilities and maintain quality preservation at lower doses with no need for ground-truth information. Currently, multiple prior studies have suggested that LDCT scans are comparable to regular CT scans in terms of providing satisfactory image quality and reduced [68, 69]. It has been reported that COVID-19 pandemic-related CT imaging features, including ground-glass opacity (GGO), reticular opacity, and parenchymal consolidations, could also be reliable observed in the LDCT protocol [68], and a prospective study compared the lesion characterization capability of standard-dose chest CT and LDCT, which showed excellent intrareader agreement [70]. The experimental results on AI method-based LDCT diagnosis show the potential to aid clinicians in the interpretation of lung cancer [71], which is expected to be implemented in COVID-19 pneumonia screening.

## AI to reduce the dose of PET

Furthermore, 18F-FDG PET/CT is a technology that can noninvasively reflect changes in metabolic and functional states in patients while evaluating and characterizing the pathogenic structures of infectious and inflammatory pulmonary conditions [72]. PET/CT is not recommended for infectious diseases due to concerns of complex procedures and excessive radiation exposure. Qin et al [28] reported that PET has the potential ability to add value to the challenge of diagnosing COVID-19 complications. Similarly, Deng et al [72] declared that the FDG PET/CT could be sensitive in monitoring inflammatory progression and treatment outcomes, they summarized evidences found FDG uptake could characterized infectious lymph node involvement better than CT imaging [73]. It is urgently needed to minimize the radiation burden and optimize imaging utilization individually. Although most AI work focuses on diagnosis analysis and treatment planning, recent work has begun to use deep learning methods to investigate dose reduction in injected tracers in PET imaging, which would bring long-term benefits to patient management in COVID-19 situations. Currently, CNNs, U-net structures, Convolutional autoencoder (CAEs), and GANs are the most widely used methods [74]. For example, Xiang et al [74] demonstrated a novel autocontext CNN method that could estimate a high-quality standard-dose PET image from relatively low-quality low-dose PET/MRI data [75]. In addition, the results show that advanced task-specific networks could obtain similar outcomes from PET-only images compared to the standard dose images [76]. Similarly, Song et al. [76] also developed deep learning methods to obtain CT-free AC PET images [77]. On the other hand, an AI-based voxelwise dosimetry prediction approach to estimate the dose post-therapy, which provides a new solution to reduce additional radiation by improving dosimetry-guided treatment planning, achieved a voxelwise mean absolute percentage error (MAPE) of 17.56% [78, 79]. However, because of the limited numbered inputting low-dose images taken into consideration, there are many challenges to provide a convincing and interpretable network.

## Conclusion

With the potential of AI, the diagnostic efficiency for coronavirus assessment via low-dose imaging methods could be improved. There is a trend to develop deep learning methods to reduce the uncertainty margin of chest radiographs and to denoise and enhance low-dose chest CT and PET to achieve equivalent diagnostic performance as imaging with the regular dose. Furthermore, an optimization algorithm should be developed in future, which balances the diagnostic benefit and radiation burden by individualizing imaging packages in different situations. In the meantime, the development of trustworthy AI algorithms requires a great number of, ideally multicenter, data sets.

## Data Availability

Data sharing not applicable to this article as no datasets were generated or analyzed during the current study.
